# Cause of death statistics in 2022 in the Republic of Korea

**DOI:** 10.12771/emj.2025.00689

**Published:** 2025-07-28

**Authors:** Jung-Hyun Oh, Juhee Seo, Hyun Jung Park

**Affiliations:** Vital Statistics Division, Statistics Korea, Daejeon, Korea

**Keywords:** Cause of death, COVID-19, Cross-sectional studies, Death certificates, Republic of Korea

## Abstract

**Purpose:**

This study aimed to describe mortality trends in the Republic of Korea in 2022 by analyzing total deaths, crude and age-standardized mortality rates, as well as age- and sex-specific patterns and changes in cause-specific mortality. The analysis updates previous reports with newly available data from 2022.

**Methods:**

A repeated cross-sectional analysis was performed using nationwide death certificate data collected through municipal administrative offices. Deaths occurring in 2022 were aggregated from reports filed over a 16-month period, spanning January 2022 to April 2023. Causes of death were classified according to the World Health Organization’s International Classification of Diseases. Quality assurance was ensured through administrative record linkage across 22 databases and validation using an independent infant mortality survey. Descriptive statistics were employed to summarize the findings.

**Results:**

In 2022, Korea recorded 372,939 deaths (the highest annual total since 1983), corresponding to a crude death rate of 727.6 per 100,000 population. This increase contributed to a net population decline of 123,751. Mortality rates rose across most age groups, with particularly marked increases among those aged 1–9 and those aged 80 or older. Coronavirus disease 2019 (COVID-19) became the third leading cause of death (31,280 deaths; 61.0 per 100,000), driven largely by the Omicron variant and heightened infection rates among older adults. Pancreatic cancer overtook stomach cancer in the mortality rankings. There were sharp increases in deaths attributed to Alzheimer’s disease and diabetes. Although deaths from intentional self-harm declined, suicide remained a significant cause of death among younger individuals.

**Conclusion:**

Korea experienced a record-high mortality rate in 2022, largely due to the impacts of COVID-19 and ongoing population aging. Notable shifts in cause-specific mortality were observed, including increases in deaths from Alzheimer’s disease, diabetes, and pancreatic cancer, underscoring evolving public health challenges.

## Introduction

### Background

In the Republic of Korea, mortality statistics are compiled in accordance with the Statistics Act and the Family Relations Registration Act. Death data, including certificates, are sourced from municipal administrative offices across the country. Cause-of-death statistics for 2014 [1], 2016 [2], 2018 [3], 2019 [4], 2020 [5], and 2021 [6] have previously been published. This report extends the analysis by incorporating newly available data from 2022.

### Objectives

This study aimed to characterize mortality in Korea in 2022, including comprehensive assessment of total death counts, the crude death rate, the age-standardized death rate (ASDR), age- and sex-specific mortality rates, and recent trends in cause-specific mortality.

## Methods

### Ethics statement

Since this study utilized publicly accessible data, Institutional Review Board approval and informed consent were not required.

### Study design

This study employed a nationwide, repeated cross-sectional design based on comprehensive death certificate data. Reporting follows the STROBE (Strengthening the Reporting of Observational Studies in Epidemiology) guidelines, available at https://www.strobe-statement.org/.

### Setting

Cause-of-death statistics in the Republic of Korea are compiled under the Statistics Act and the Act on the Registration of Family Relations. Data are collected using death notification forms and medical death certificates submitted to administrative offices nationwide. Deaths that occurred in 2022 were aggregated from reports filed over a 16-month period, spanning January 2022 through April 2023. All notifications and certificates were coded for the underlying cause of death according to the World Health Organization’s International Classification of Diseases guidelines.

### Participants (subjects)

Subjects included all death certificates for persons who died in Korea in 2022. While foreigners who died in Korea were documented separately, they were excluded from this analysis.

### Data source/measurement

The data collection and analytical methods were consistent with those used in earlier cause-of-death studies, ranging from the 2016 report [2] through the 2018–2021 series [3-6]. For this analysis, all death certificates issued in 2022 for Korean residents of the Republic of Korea served as the primary data source [7].

To enhance the reliability of the mortality figures, 2 additional quality-assurance procedures were implemented. First, an independent infant mortality survey was conducted, since infant deaths are often underreported, by canvassing medical institutions and collecting cremation records from all crematoriums nationwide. Second, administrative record linkage was performed to validate the accuracy of recorded causes of death. This process involved cross-referencing each case against 22 distinct administrative databases, including national health insurance claims, the national cancer registry, police investigation files, autopsy reports, and other relevant records.

### Variables

All causes of death were included as variables in the analysis.

### Bias

No selection bias was present, as data from all subjects were included.

### Study size

The entire population of the Republic of Korea was included; therefore, sample size estimation was unnecessary.

### Statistical methods

Data were summarized using descriptive statistics only. No analytic statistical tests were performed.

## Results

### Number of deaths and crude mortality rate

**Fig. 1.**
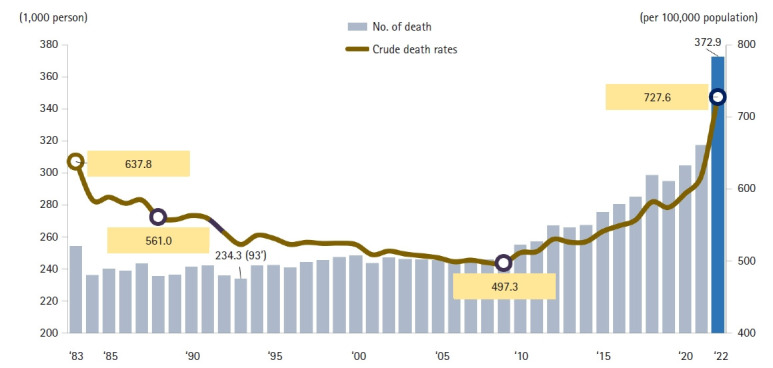
The annual number of deaths and the crude death rate from 1983 to 2022 in Korea.

In 2022, the total number of deaths in the Republic of Korea was 372,939, representing an increase of 55,259 deaths (17.4%) compared with 2021 (Fig. 1, Supplement 1). Male deaths numbered 196,465, which is an increase of 24,498 (14.2%) from the previous year, while female deaths totaled 176,474, up by 30,761 (21.1%). The average daily number of deaths rose to 1,022, which is 152 more than in the preceding year. The crude mortality rate (deaths per 100,000 population) was 727.6, reflecting an increase of 108.7 (17.6%) from 2021. Specifically, the male crude mortality rate reached 769.2 per 100,000 (an increase of 97.2, or 14.5%), while the female crude mortality rate rose to 686.2 per 100,000 (an increase of 120.2, or 21.2%). The male-to-female mortality ratio was 1.12, indicating that men’s mortality rate was 1.12 times that of women. Both the total number of deaths and the crude mortality rate were the highest since national statistics were first compiled. The ASDR, which adjusts for age distribution, was 372.9, up by 55.3 from 2021 (Fig. 1, Supplement 1).

### Deaths by sex and age group

**Fig. 2.**
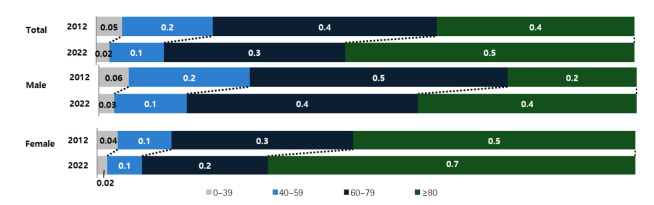
Trends in sex- and age-specific proportions of deaths, 2012 vs. 2022 in Korea.

**Fig. 3.**
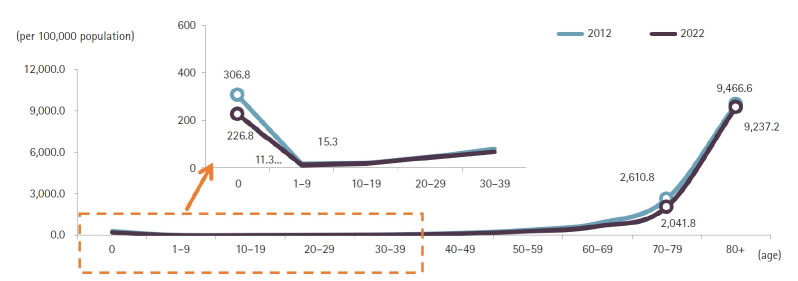
Age-specific mortality rates by sex, 2012, 2021, and 2022 in Korea.

Compared with 2021, the number of deaths increased most substantially among children aged 1–9 years (33.8%), adults aged 80 years and older (26.3%), those in their 70s (11.3%), and those in their 60s (10.2%) (Figs. 2, 3, Supplements 2, 3). Individuals aged 80 years and older accounted for 53.8% of all deaths in 2022, marking a 17.0 percentage-point increase from a decade earlier. Among male decedents, 40.7% were aged 80 or older—an increase of 16.7 percentage points over the past 10 years—while 68.3% of female decedents were in this age group, a 15.9 percentage-point rise. The male-to-female ratio of death counts peaked in the fifth and sixth decades of life, at 2.6. Age-specific mortality rates were lowest among children aged 1–9 years (11.3 per 100,000) and highest among those aged 80 years and older (9,237.2 per 100,000). The male crude mortality rate increased by 14.5% to 769.2 per 100,000, and the female rate rose by 21.2% to 686.2 per 100,000. Among males, age-specific mortality rates rose in the 1–9 and 10–19-year cohorts and in all age groups over 30. Among females, rates increased in the 1–9, 10–19, 20–29, and all age groups over 40. In every age stratum, males had higher mortality rates than females, with the greatest disparity in the sixth decade, where the male rate was 2.7 times that of females.

### Leading causes of death

**Fig. 4.**
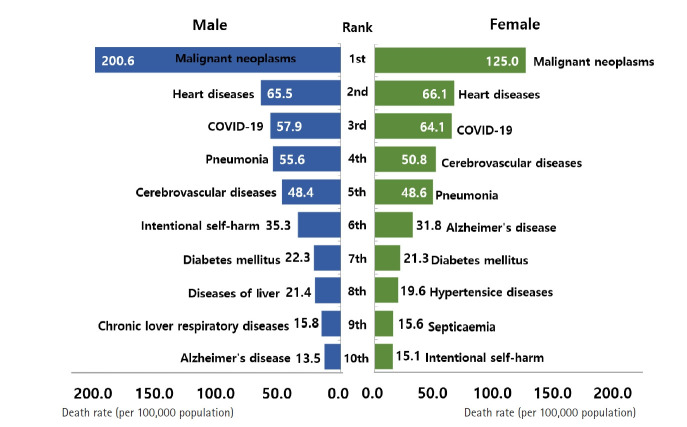
The 10 leading causes of death by sex in 2021 in Korea.

In 2022, the top 10 causes of death were malignant neoplasms (cancer), heart disease, coronavirus disease 2019 (COVID‑19), pneumonia, cerebrovascular disease, intentional self‑harm, Alzheimer’s disease, diabetes mellitus, hypertensive diseases, and liver disease (Fig. 4, Supplement 4). These 10 causes accounted for 67.4% of all deaths. The top 3—cancer, heart disease, and COVID-19—comprised 39.8% of total mortality, a 3.4 percentage-point decrease from 2021. COVID-19 appeared in the top 10 for the first time, ranking third. Malignant neoplasms and heart disease remained the leading causes by mortality rate, while the mortality rate for hypertensive diseases rose by 2.9 per 100,000 to 15.1 per 100,000. Compared to a decade earlier, pneumonia, Alzheimer’s disease, and hypertensive diseases rose in ranking.

Among males, the 10 leading causes, in order, were malignant neoplasms, heart disease, COVID‑19, pneumonia, cerebrovascular disease, intentional self‑harm, diabetes mellitus, liver disease, chronic lower respiratory disease, and Alzheimer’s disease. Male mortality rates exceeded those of females for cancer, pneumonia, intentional self‑harm, diabetes mellitus, liver disease, and chronic lower respiratory disease. Among females, the top 10 were malignant neoplasms, heart disease, COVID‑19, cerebrovascular disease, pneumonia, Alzheimer’s disease, diabetes mellitus, hypertensive diseases, sepsis, and intentional self‑harm. Females had higher mortality rates than males for heart disease, COVID‑19, cerebrovascular disease, Alzheimer’s disease, hypertensive diseases, and sepsis. For both sexes, malignant neoplasms were the leading cause, with the male cancer mortality rate 1.6 times that of females. COVID‑19 and Alzheimer’s disease appeared in the male top 10 for the first time, while the ranking of intentional self‑harm fell for both sexes (males: 5th→6th; females: 7th→10th).

By age group, cancer was the leading cause of death among individuals aged 1–9 years and those aged 40 years and above; it was the second leading cause in the 10–19, 20–29, and 30–39 cohorts. Heart disease ranked second among those in their 60s and in all age groups except teenagers, consistently appearing in the top 5. COVID‑19 ranked second for those aged 70 and older, third among children aged 1–9 years, and fourth in the 10–19 and 60–69 age groups. Pneumonia ranked fourth among those aged 80 and older and fifth in the 70s, highlighting increased risk in the oldest cohorts. Cerebrovascular disease was third in the 60s, fourth in the 70s, and fifth in the 40s, 50s, and those 80 and over. Liver disease ranked highest—third—among those in their 40s, and was fourth among those in their 30s and 50s. Intentional self‑harm was the leading cause of death in teens through the 30s, second in the 40s and 50s, and fifth in the 60s.

### Trends in cause‑specific mortality rates

#### Overall trends

**Fig. 5.**
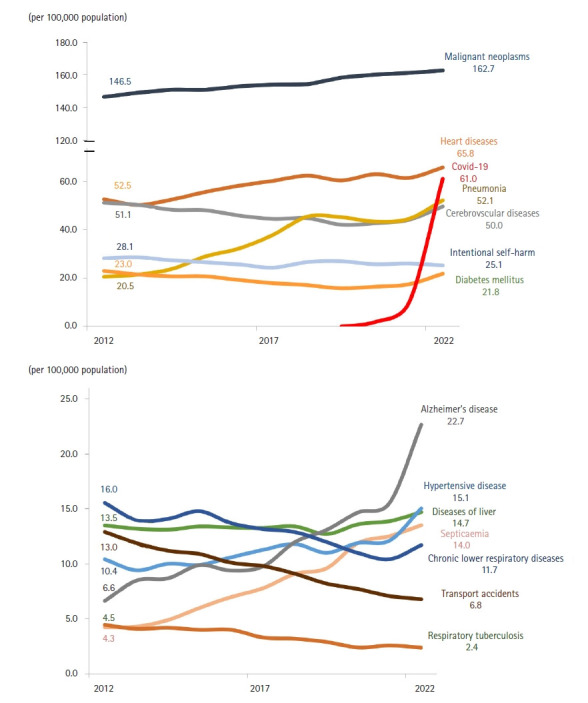
Mortality rates trends for major causes of death in Korea.

Compared with 2021, the most substantial relative increases in mortality rates (per 100,000 population) were observed for COVID‑19 (522.8%), Alzheimer’s disease (45.6%), diabetes mellitus (24.9%), hypertensive diseases (24.2%), pneumonia (17.3%), and cerebrovascular disease (12.6%) (Fig. 5, Supplement 5). In contrast, marked declines were noted for respiratory tuberculosis (–7.5%), transport accidents (–4.1%), and intentional self‑harm (–3.2%). Over the past decade, the most pronounced long‑term increases have occurred in Alzheimer’s disease (241.2%), sepsis (218.0%), pneumonia (154.4%), hypertensive diseases (44.7%), and heart disease (25.2%). Conversely, substantial long‑term declines have been recorded for transport accidents (–47.6%), respiratory tuberculosis (–46.5%), chronic lower respiratory diseases (–24.7%), intentional self‑harm (–10.5%), and diabetes mellitus (–5.0%).

#### Malignant neoplasms

**Fig. 6.**
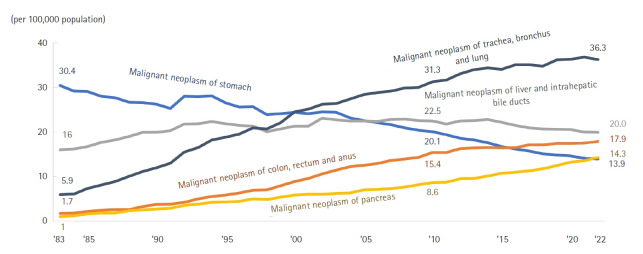
Trends in mortality from malignant neoplasms by organ site from 1983 to 2022 in Korea.

The overall cancer mortality rate was 162.7 per 100,000, representing a 1.0% increase (1.6 per 100,000) from 2021. Lung cancer (36.3 per 100,000) remained the leading cause of cancer death, followed by liver cancer (19.9 per 100,000), colorectal cancer (17.9 per 100,000), pancreatic cancer (14.3 per 100,000), and stomach cancer (13.9 per 100,000) (Fig. 6, Supplement 6). Compared with the previous year, mortality rates increased for pancreatic cancer (5.8%), brain cancer (5.5%), and breast cancer (5.0%), but declined for uterine cancer (–4.3%), lung cancer (–1.5%), and stomach cancer (–1.3%). The cancer mortality rate for males (200.6 per 100,000) was 1.6 times higher than that for females (125.0 per 100,000). Among men, the highest mortality rates were recorded for lung cancer (53.7 per 100,000), liver cancer (29.1 per 100,000), and colorectal cancer (20.6 per 100,000); among women, the most fatal cancers were lung (18.9 per 100,000), colorectal (15.2 per 100,000), and pancreatic cancer (13.7 per 100,000). The most pronounced sex-specific disparity was seen in esophageal cancer, with a male-to-female ratio of 9.1, followed by lung cancer (2.8) and liver cancer (2.7). From 2021 to 2022, overall cancer mortality rates for both sexes increased by 1.6 per 100,000 (0.8% for men; 1.3% for women). Over the past decade, mortality rates have risen for pancreatic, lung, prostate, and breast cancers, while rates for stomach and liver cancers have declined. By age group, brain cancer was the leading cause among teenagers, leukemia in the 20s, stomach cancer in the 30s, breast cancer in the 40s, liver cancer in the 50s, and lung cancer among those aged 60 and above.

### Circulatory system diseases

**Fig. 7.**
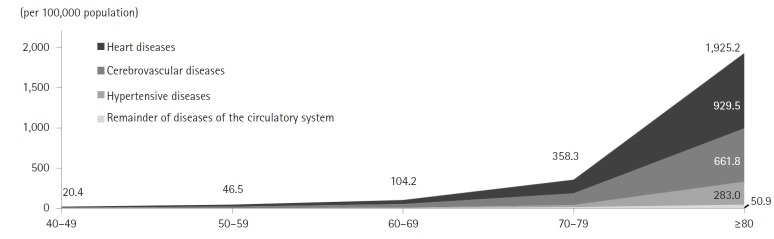
The mortality rate due to circulatory system diseases by age in 2022 in Korea.

The mortality rate for circulatory system diseases was 134.7 per 100,000, with heart disease accounting for 65.8 per 100,000, cerebrovascular disease for 49.6 per 100,000, and hypertensive diseases for 15.1 per 100,000 (Fig. 7, Supplement 7). Compared with the previous year, rates increased for hypertensive diseases by 24.2%, cerebrovascular disease by 12.6%, and heart disease by 7.0%. Within heart disease, “other heart diseases” had the highest rate at 37.0 per 100,000. Female mortality from circulatory diseases (140.6 per 100,000) was 1.1 times higher than that of males (128.7 per 100,000). While women exhibited higher mortality for hypertensive and cerebrovascular diseases, men had a greater ischemic heart disease mortality rate (33.2 per 100,000 versus 24.3 per 100,000 for women). From 2021 to 2022, circulatory disease mortality increased by 12.6 per 100,000 (10.8%) among men and 13.8 per 100,000 (10.9%) among women. Mortality from circulatory diseases rose consistently with age, and in every age group, heart disease, cerebrovascular disease, and hypertensive diseases were the top 3 causes. Among individuals in their 40s through 60s, ischemic heart disease predominated, whereas in teenagers, those in their 20s, and those aged 70 and above, other heart diseases were most frequent.

#### External causes of death (including accidents)

**Fig. 8.**
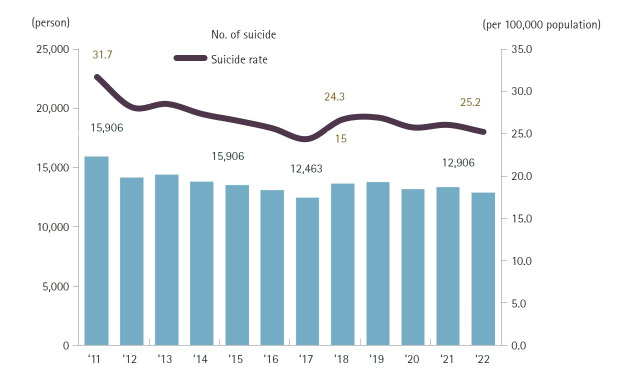
Number of deaths and mortality rate due to intentional self-harm, 2011–2022 in Korea.

**Fig. 9.**
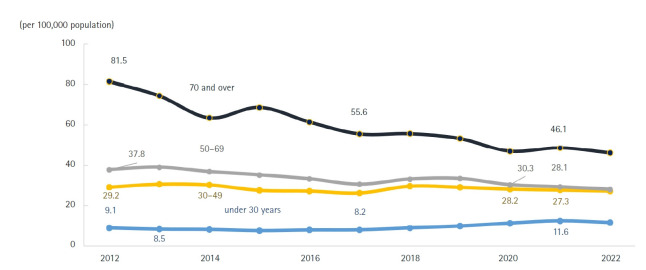
Age-specific intentional self-harm rates, 2012–2022 in Korea.

Non-disease external causes accounted for 7.2% of all deaths (26,688 cases), down 1.1 percentage points from 8.2% in 2021 (Figs. 8, 9, Supplements 8, 9). The mortality rate for external causes was 52.1 per 100,000, a 2.2% increase from the prior year. The leading external causes were suicide (25.2 per 100,000), transport accidents (6.8 per 100,000), and falls (5.3 per 100,000). Mortality rates declined for homicide (–10.0%), transport accidents (–4.1%), and suicide (–3.2%), but rose for fire-related accidents (9.2%), poisoning (4.7%), and drowning (2.5%). The external-cause mortality rate for males (71.4 per 100,000) was 2.2 times that for females (32.9 per 100,000). The highest male-to-female ratios were observed for drowning (3.2), transport accidents (3.0), and falls (2.6). By age, homicide (such as abandonment) (4.0 per 100,000), falls (1.2 per 100,000), and transport accidents (0.4 per 100,000) were most common among infants (0 years); in children aged 1–9 years, homicide (0.6) and equal rates (0.4) for transport, falls, and drowning accidents were seen; among individuals aged 10–79 years, suicide and transport accidents were most frequent; and in those aged 80 and older, suicide (60.6), falls (42.8), and transport accidents (29.3) per 100,000 were predominant.

#### Intentional self-harm trends

In 2022, there were 12,906 deaths by intentional self-harm, a decrease of 446 cases (3.3%) compared to 2021. Monthly declines were especially notable in March (–16.0%), June (–15.3%), and February (–13.1%). The average daily number of suicides was 35.4. The mortality rate for intentional self-harm was 25.2 per 100,000, a reduction of 0.8 (3.2%) from the previous year. Rates increased in the 40s (2.5%) and among teenagers (0.6%), but decreased in the 70s (–9.6%), 20s (–9.2%), 30s (–7.2%), 60s (–4.7%), 50s (–3.6%), and those aged 80 and above (–1.1%). Male intentional self-harm mortality (35.3 per 100,000) was 2.3 times higher than that for females (15.1 per 100,000). Both sexes experienced declines (males –1.7%; females –6.4%), with the lowest male-to-female ratio in the teenage group (1.1) and the highest in those aged 80 and older (3.8). Intentional self-harm remained the leading cause of death among those aged 10–39, and the second leading cause among those in their 40s and 50s.

### Alcohol‑related mortality

**Fig. 10.**
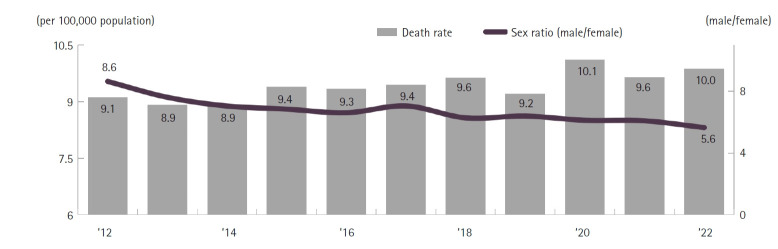
Trends in the sex ratio of alcohol-related mortality, 2012–2022 in Korea.

In 2022, there were 5,033 deaths attributable to alcohol, averaging 13.8 deaths per day and representing an increase of 105 deaths compared to 2021 (Fig. 10, Supplement 10). The alcohol-related mortality rate was 9.8 per 100,000, marking a 2.3% increase from the previous year. Male alcohol-related mortality rose in the 20s, 30s, 50s, and 70s, while female rates increased in all age groups except those in their 30s. The male mortality rate (16.7 per 100,000) was 5.7 times higher than that for females (3.0 per 100,000). Alcohol-related mortality rates increased steadily after the 30s, peaking in the 50s before declining.

### Dementia‑related mortality

**Fig. 11.**
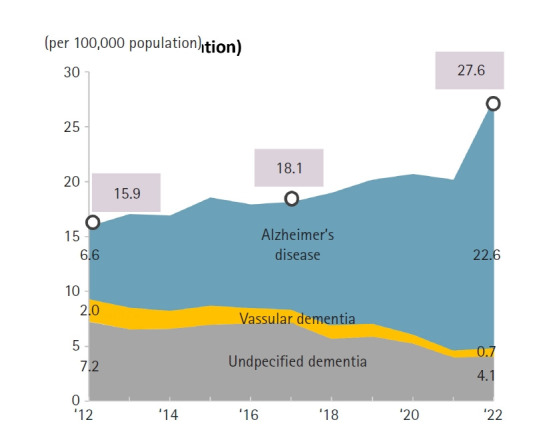
Trends in mortality rates due to dementia by cause, 2012–2022 in Korea.

Deaths attributed to dementia reached 14,136 in 2022, representing a 36.6% increase compared to 2021. The dementia mortality rate was 27.6 per 100,000, an increase of 7.4 (36.8%) (Fig. 11, Supplement 11). Female dementia mortality (38.0 per 100,000) was 2.2 times that of males (17.1 per 100,000). Both sexes experienced substantial year-over-year increases in dementia-related mortality (males 32.9%; females 38.5%).

### COVID‑19 mortality

**Fig. 12.**
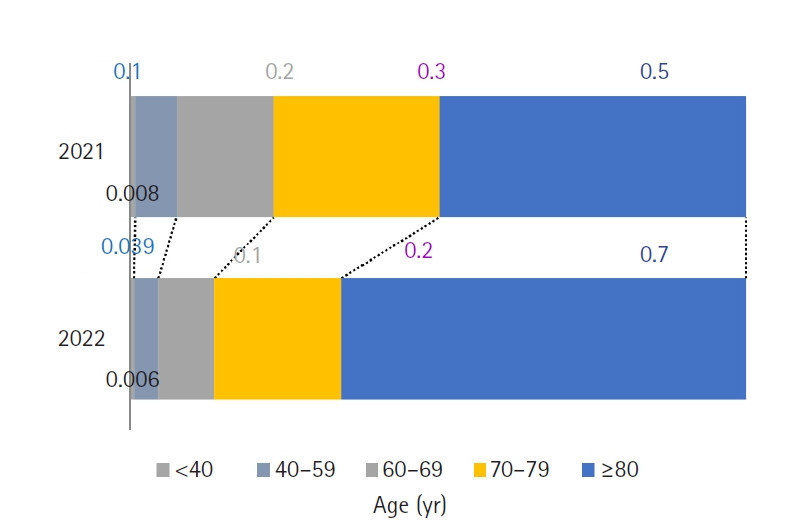
COVID-19-related deaths per 100,000 population by age and sex, 2021 and 2022 in Korea.

In 2022, COVID-19 was responsible for 31,280 deaths, accounting for 8.4% of all fatalities. This number surpassed the 26,250 COVID-19 deaths recorded in 2021 (Fig. 12, Supplement 12). The COVID-19 mortality rate reached 61.0 per 100,000, representing a 51.2-point (522.8%) increase over the previous year. Mortality rose sharply with age, peaking at 946.0 per 100,000 among individuals aged 80 years and older. Monthly COVID-19 deaths were highest in March (10,955), followed by April (6,875).

## Discussion

The year 2022 marked a significant demographic shift for Korea, with the highest number of recorded deaths since 1983, totaling 372,937, an increase of 55,259 compared to the previous year. Simultaneously, the crude mortality rate reached an all-time high of 727.6 per 100,000 population (Fig. 1). This substantial rise in mortality, combined with a birthrate of 249,186 in 2022, resulted in a net population decrease of 123,751 [7]. Notably, this decline was more than double the decrease observed in 2021 (57,080). Korea’s total fertility rate in 2022 was just 0.78, the lowest in the world, suggesting that population decline will likely continue unless there is a dramatic increase in birth rates.

### Age-specific mortality trends

Analysis of age-specific mortality rates per 100,000 population revealed a decrease of 6.0 deaths in the 0-year-old cohort and a reduction of 0.8 deaths among individuals in their 30s. In contrast, mortality rates rose in all other age groups. The most pronounced increase occurred in the 1–9 age group, with a rise of 42.0 deaths, while those aged 80 and above experienced an increase of 17.7 deaths (Fig. 3).

### Leading causes of death

Among the causes of death, COVID-19-related fatalities rose sharply to 31,280 in 2022, a substantial jump from 5,030 in 2021. This increase resulted in a COVID-19 mortality rate of 61.0 per 100,000, accounting for 8.4% of all deaths and making it the third leading cause of death for both men and women, underscoring the rapid escalation of pandemic-related mortality (Fig. 4). The surge in COVID-19 deaths is primarily attributed to the high transmissibility of the Omicron variant and widespread community infection, particularly among the elderly.

The Omicron (BA.1) variant became the dominant strain in Korea in early 2022 [8], resulting in a dramatic surge in confirmed cases after January, with a peak of 9,959,368 confirmed cases in March. Notably, 55.4% (14,735/26,593) of all COVID-19 deaths in 2022 occurred in March and April—a period characterized by increased infections among older adults [9]. Although Korea achieved high vaccination rates—as of May 31, 2022, the proportions of the Korean population with complete vaccination and an additional booster shot were 86.8% and 66.9%, respectively, making Korea one of the countries with the highest vaccination rates worldwide [10]—a considerable number of deaths still occurred, particularly in highly vulnerable groups such as those over 80 years old, who experienced elevated fatality rates in breakthrough infections due to their relatively compromised immune systems [11]. During 2020–2021, Korea successfully suppressed community transmission through stringent containment measures such as social distancing. However, all social distancing restrictions were lifted in mid-April 2022 after a confirmed decline in the epidemic curve. It is important to note that this decision was made after the Omicron surge had peaked (mid-March), and that targeted prevention strategies for high-risk groups continued even after the lifting of restrictions.

Alzheimer’s disease has shown a steady increase since entering the top 10 causes of death in 2018, rising from 15.6 to 22.7 deaths per 100,000 population by 2022 (Fig. 5).

Among the various causes of death, diabetes surpassed liver disease in the rankings for men. Traffic accidents and septicemia also fell out of the top 10 causes. For women, intentional self-harm declined in ranking relative to hypertensive diseases and septicemia, settling at the 10th position (Figs. 4, 8). The ongoing decrease in intentional self-harm among women compared to men remains difficult to explain and requires further observation to determine if this trend will persist.

### Cancer mortality trends

Regarding cancer-related deaths, the rankings for lung cancer, liver cancer, and colorectal cancer remained unchanged from 2021. However, pancreatic cancer notably surpassed stomach cancer for the first time, rising to fourth place (Fig. 6). This pattern suggests a continuing decrease in stomach cancer deaths and a sustained increase in pancreatic cancer deaths in the future. While liver cancer deaths are expected to decline, colorectal cancer mortality is projected to remain stable. The decline in stomach cancer mortality can be attributed to the inclusion of gastroscopy in national health screening programs and increased screening participation, which have promoted earlier detection and shifts in dietary habits [12]. Similarly, the inclusion of occult blood tests in national screening and the widespread adoption of colonoscopy have helped prevent an increase in colorectal cancer mortality through early detection [13]. Lung cancer deaths have shown a slight decrease (Fig. 6), which may be attributable to biennial chest X-ray screening in national health check-ups. The application of artificial intelligence to chest X-ray interpretation is expected to further enable earlier lung cancer detection in the future [14].

### Age as a determinant of mortality

Age is a primary determinant of mortality in South Korea. Older adults are especially vulnerable to chronic diseases such as diabetes and hypertension, as well as infectious diseases like septicemia and pneumonia. In this population, diminished immune function amplifies the burden of these illnesses. Notably, the prevalence of diabetes increases with age and is a major contributor to rising mortality among those aged 70 and above. The diabetes-related mortality rate per 100,000 population has climbed in recent years, from 16.5 in 2020 to 17.5 in 2021, and sharply to 21.8 in 2022 (Fig. 5).

### Declining traffic accident fatalities and stable alcohol-related deaths

Traffic accident fatalities have shown a consistent decline (Fig. 5), a trend attributed to interventions such as the introduction of safety regulations in child protection zones in 1995, amendments to the Road Traffic Act in 2020, stricter penalties for drunk driving, and heightened public awareness of road safety. Alcohol-related deaths have remained relatively stable at 9.9 per 100,000 population, only a slight change from 9.6 in 2021 (Fig. 10). Without the implementation of robust national prohibition policies—such as banning alcohol consumption scenes in media, enforcing stricter license suspensions for drunk driving, and imposing harsher legal penalties for alcohol-related accidents—significant reductions in alcohol-related mortality are unlikely in the near term.

### Conclusion

Korea experienced a marked increase in overall mortality in 2022, reaching unprecedented levels, mainly due to the emergence of COVID-19 as the third leading cause of death and the impact of an aging population. Notable shifts in mortality patterns included pancreatic cancer surpassing stomach cancer and significant increases in deaths related to Alzheimer’s disease and diabetes.

## Supplementary materials

Supplementary materials are available from Harvard Dataverse: https://doi.org/10.7910/DVN/DPXEXD

Supplement 1. The annual number of deaths and the crude death rate in Korea from 1983 to 2022.

Supplement 2. Trends in sex- and age-specific proportions of deaths, 2012 vs. 2022 in Korea.

Supplement 3. Age-specific mortality rates by sex, 2012, 2021, and 2022 in Korea.

Supplement 4. The 10 leading causes of death by sex in 2021 in Korea.

Supplement 5. Mortality rates trends for major causes of death in Korea.

Supplement 6. Trends in mortality from malignant neoplasms by organ site from 1983 to 2022 in Korea.

Supplement 7. The mortality rate due to circulatory system diseases by age in 2022 in Korea.

Supplement 8. Number of deaths and mortality rate due to intentional self-harm, 2011–2022 in Korea.

Supplement 9. Age-specific intentional self-harm rates, 2012–2022 in Korea.

Supplement 10. Trends in the sex ratio of alcohol-related mortality, 2012–2022 in Korea.

Supplement 11. Trends in mortality rates due to dementia by cause, 2012–2022 in Korea.

Supplement 12. COVID-19-related deaths per 100,000 population by age and sex, 2021 and 2022 in Korea.
